# Impact of parylene coating on heating performance of intravenous fluid warmer: a bench study

**DOI:** 10.1186/s12871-022-01585-w

**Published:** 2022-02-10

**Authors:** Danielle K. Bayoro, Herman Groepenhoff, Daniel Hoolihan, Edward A. Rose, Michael J. Pedro, Andreas D. Waldmann

**Affiliations:** Department of Medical Affairs, Vyaire Medical, 26125 North Riverwoods Blvd, Mettawa, IL 60045 USA

**Keywords:** Hypothermia, Perioperative care, Parenteral therapy, Intravenous fluid warmer, Fluid resuscitation

## Abstract

**Background:**

Perioperative hypothermia is a common occurrence, particularly with the elderly and pediatric age groups. Hypothermia is associated with an increased risk of perioperative complications. One method of preventing hypothermia is warming the infused fluids given during surgery. The enFlow™ intravenous fluid warmer has recently been reintroduced with a parylene coating on its heating blocks. In this paper, we evaluated the impact of the parylene coating on the new enFlow’s fluid warming capacity.

**Methods:**

Six coated and six uncoated enFlow cartridges were used. A solution of 10% propylene glycol and 90% distilled H_2_O was infused into each heating cartridge at flow rates of 2, 10, 50, 150, and 200 ml/min. The infused fluid temperature was set at 4 °C, 20 °C, and 37 °C. Output temperature was recorded at each level. Data for analysis was derived from 18 runs at each flow rate (six cartridges at three temperatures).

**Results:**

The parylene coated fluid warming cartridge delivered very stable output of 40 °C temperatures at flow rates of 2, 10, and 50 ml/min regardless of the temperature of the infusate. At higher flow rates, the cartridges were not able to achieve the target temperature with the colder fluid. Both cartridges performed with similar efficacy across all flow rates at all temperatures.

**Conclusions:**

At low flow rates, the parylene coated enFlow cartridges was comparable to the original uncoated cartridges. At higher flow rates, the coated and uncoated cartridges were not able to achieve the target temperature. The parylene coating on the aluminum heating blocks of the new enFlow intravenous fluid warmer does not negatively affect its performance compared to the uncoated model.

## Introduction

Between 25 and 90% of all patients who undergo elective surgery experience hypothermia, defined as a body temperature below 36 °C [1]. Anesthesia (regional and general) can interfere with the body’s thermoregulatory processes [2]. The anesthetic drugs themselves can create challenges for temperature control either centrally or peripherally. The risk of hypothermia is influenced by the temperature of the operating room suite, the temperature of stock fluids and blood products, and the rate of administration of intravenous infusates [3].

Hypothermia is associated with an increased risk of complications, some of which can be severe [4, 5]. Several authors have reported an association with poor wound healing, cardiac dysrhythmias, and increased bleeding [4, 6, 7]. Even mild hypothermia of only 1-3 °C is associated with increased complications such as delayed wound healing, ventricular tachycardia, poor anesthetic drug clearance, coagulopathy, and susceptibility to infection [6, 8, 9]. In contrast, maintenance of normothermia reduces hospital costs and death rates [10].

Numerous methods have been devised to prevent inadvertent hypothermia [2, 11]. Surgical personnel typically use warming blankets [12], warm the surgical suite, warm the inspired air in the ventilator, utilize cabinet storage warmers for infusates [13], and reduce the rate of infusion of fluids and blood products. These and numerous other techniques have been described as the prevention of hypothermia has gained increased attention [11, 14–16].

Current guidelines recommend incorporating a method of warming infused fluids during surgery [3, 11, 15, 17]. The heating performance of several fluid warmers has been evaluated in the past [18–20].

Kim et al. studied the effect of higher flow rates of fluid on output temperature over time [21]. In their study set-up, they tested pressurized isotonic saline at 5 °C and 20 °C, flowing at 30, 50 and 100 ml/min for 6 min. They found that the ThermoSens® (Sewoon Medical Company, Seoul, Korea) and buddy light™ (Belmont Instrument Corporation, Billerica, MA, USA) underperformed at higher flow rates compared to the uncoated enFlow™ (Vyaire Medical, Mettawa, IL, USA). Zoremba [22] and colleagues found similar performance results using ice-cold saline comparing the Fluido Compact® (The 37° Company, Amersfoort, Netherlands) and the Thermosens® (Barkey, Leopoldshöhe, Germany) fluid warmers to the uncoated enFlow. Room temperature (24.4 °C) fluid and chilled (6 °C) fluid were run through the warmers at 25, 50, 75, and 100 ml/min. At a flow rate of 25 ml/min and 50 ml/min, the outlet temperature from enFlow was significantly higher (p < 0.01) than the output temperatures of Fluido Compact and Thermosens warmers. Another study by Xu et al. used isotonic saline at infusion drip rates of 3, 4, 5, 6, 7, 8, 10, and 17.5 ml/min adjusted to room temperatures of 20 °C, 22 °C, and 24 °C. They measured temperatures at the outlet of both the 3 M Ranger™ (3 M) dry heat transfer heater and the FT2800™ (Keewell Medical Technology, China) coaxial coil heater. Output temperatures were significantly affected by room temperature and flow rates [23]. Even at only 17.5 ml/min, neither unit tested was able to reach its target temperature as flow rates increased.

Intravenous fluid warmers based on aluminum heating blocks have been troubled with aluminum leaching into the fluid, leading to concerns about aluminum toxicity [24–27]. The Food and Drug Administration recently issued a letter to health care providers in this regard, mentioning that multiple fluid warmers have been restricted or recalled [28]. In response to this issue, the enFlow® intravenous fluid warmer (Vyaire Medical, Mettawa, IL) was recently redesigned to include a parylene coating over the heating block [26]. As previous studies only evaluated the heating performance of the uncoated enFlow system, currently no published data are available on the performance of the new parylene-coated enFlow cartridge.

Heat transfer from the aluminum block to the passing fluid takes place through forced convection. The equation is expressed as Newton’s Law of Cooling:

$$Q={h}_cA\ dT$$

where:

Q is the heat transfer to the liquid in Watts.

h_c_ is the convective heat transfer coefficient for the process and is a function of both the liquid velocity and liquid properties for a given fluid.

A is the area of plate in contact with the liquid in the cartridge.

and dT is the temperature difference between the plate and the fluid.

The heat transfer coefficient in forced convection for aluminum falls between (59-64)w/m^2^.K [29]. This coefficient varies according to the velocity and physical properties of the current of fluid over the block. The overall heat transfer equation is affected by a variety of factors including flow rate, cartridge geometry, turbulence, fluid viscosity, temperature of the heated plate, and temperature difference of input temperature compared to the heated plate. Parylene as a thermal insulator adds another layer of complexity. Heating the aluminum behind the parylene represents heating via conduction and varies by the thickness of the parylene. Therefore, we compared the uncoated and coated models of the enFlow fluid warmer at varying fluid rates at different fluid temperatures to evaluate if the parylene coating is an acceptable option to overcome the described issues with aluminium leaching, without impacting the heating performance of enFlow cartridge.

## Methods

**Fig. 1:**
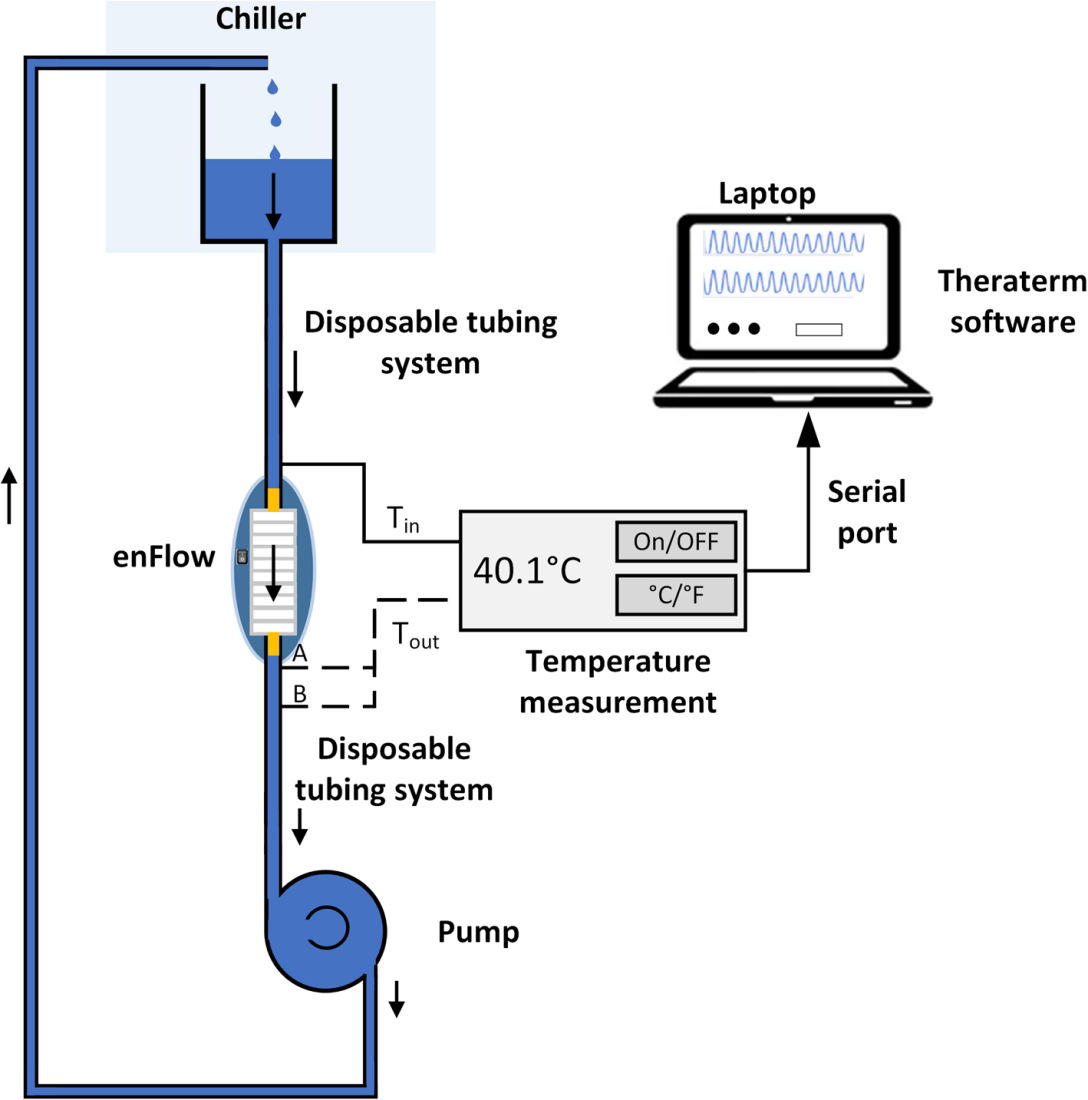
Experimental setup. Warmed or chilled fluid was run through the warming cartridge at a rate determined by the peristaltic pump. Temperature was measured before and after the warming cartridge. **A** denotes the location of the temperature sensor for the parylene-coated enFlow experiments (0.635 cm); **B** denotes the location of the temperature probe for the uncoated enFlow experiments (between 10 and 20 cm). Data was routed to a laptop computer for analysis. Fluid was recycled back into the chiller after infusion

In this study, we evaluated the efficacy of two fluid warmers (parylene-coated and uncoated enFlow cartridges) under several conditions. A solution with thermal properties equivalent to 5% dextrose was produced using 10% propylene glycol (PG) and 90% distilled H_2_O. A 10 L capacity chiller tank (Polystat 3C15++; Cole-Palmer; Vernon Hills, IL) was filled with the 10% PG solution, and the solution was maintained in one of three different temperature ranges: cold (4 °C), normal (around 20 °C), and warm (more than 35 °C). The conditioned samples were then maintained in the temperature range using the chiller tank. Five different flow rates were tested in this study: 2 ml/min, 10 ml/min, 50 ml/min, 150 ml/min, and 200 ml/min. A pump (Masterflex L/S® Peristaltic Pump, Masterflex, Vernon Hills, IL, and Easy-Load® II Pump Head, Cole-Palmer; Vernon Hills, IL) was used to generate the different flow rates. The peristaltic pump was calibrated each time the tubing or cartridge was changed using a volume per unit of time procedure, according to the pump manual instructions. A standard non-insulated extension tubing set (Masterflex L/S® Higher Performance Precision Pump Tubing; Masterflex, Vernon Hills, IL) was connected at the fluid warmer outlet. Measurements were performed in a laboratory setting at 21-24 °C room temperature. The fluid temperature was measured at the input (T_in_) and the output (T_out_) of the enFlow cartridge (see Fig. 1) using a two-channel thermometer (Thermistors: Omega Engineering, part number TJ36-CAXL-020 U-6, Norwalk, CT; and USB-TC DAQ, Measurement Computing Corporation, Norton, MA). The temperature was measured within a distance of 0.635 cm from the warming cartridge for the parylene-coated enFlow cartridge and between 10 and 20 cm from the warming cartridge for the uncoated enFlow warming cartridge. After starting each trial, the temperatures at the two positions (T_in_, T_out_) were recorded by a laptop computer with data acquisition terminal using Tera Term (Tera Term Project, open source).

The parylene-coated enFlow cartridge begins delivering target temperatures within seconds of activation of the heating unit. Starting at 2 min after the fluid infusion was begun to allow for equilibration, the input and output fluid temperatures were recorded and averaged over 15 s. The measurements were repeated for six different coated and uncoated enFlow cartridges to evaluate inter-device variability. A total of 18 data points for T_in_ and T_out_ were obtained (six cartridges at three temperatures each) for each of five flow rates and used for the statistical analysis (total 90 data points for each model of enFlow cartridge).

Statistical analysis and graphical presentation were performed using GraphPadPrism 9.2.0 (GraphPad Software, San Diego, CA 92108).

## Results

**Fig. 2:**
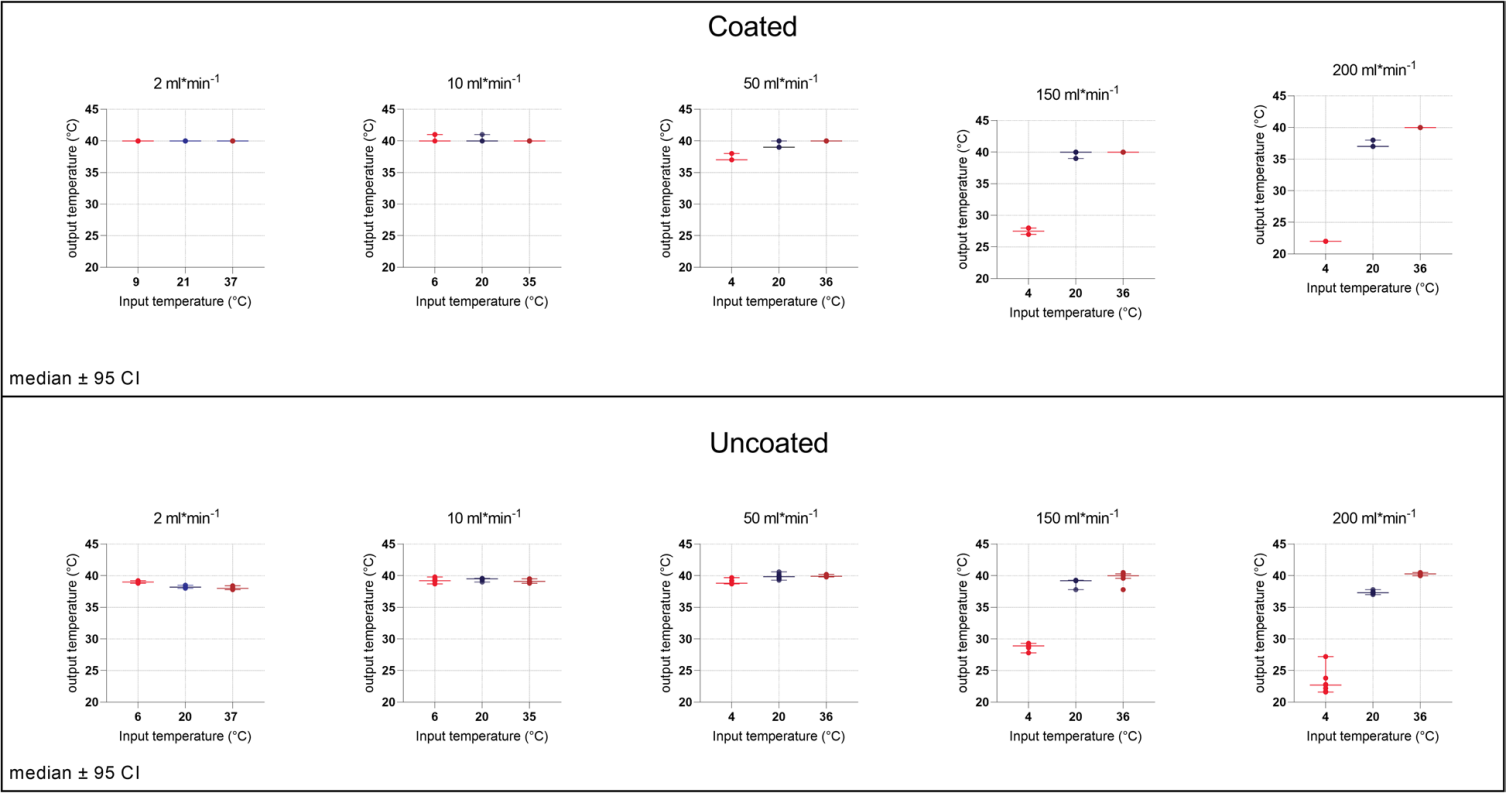
Comparison of input vs. output temperature recordings with parylene-coated fluid warmer cartridge (**A**) and uncoated fluid warmer cartridge (**B**). Lines indicate medians ±95% confidence intervals

Figure 2 shows plots of input temperature vs. output temperature at each temperature and flow rate. The mean value of T_out_ is plotted with error bars representing the standard error of the mean. The parylene-coated fluid warming cartridge delivered target output temperatures at flow rates of 2, 10, and 50 ml/min regardless of the temperature of the infusate. At the lowest flow rates (2 ml/min and 10 ml/min), both enFlow cartridges achieved their target set temperature for all fluid temperatures.

At the higher flow rates (150 and 200 ml/min), the heating performance was also similar between both warmers. Output temperatures were clearly impacted by the input temperatures at 150 and 200 ml/min. With an input temperature of 4 °C, the parylene-coated enFlow increased the temperature of the fluid by 23.5 °C at 150 ml/min and by 18 °C at 200 ml/min. With an input temperature of 20 °C, the parylene-coated enFlow increased the temperature of the fluid by 19.7 °C (to 39.8 °C) at 150 ml/min and by 17.3 °C (to 36.7 °C) at 200 ml/min. With an input temperature of 37 °C, the parylene-coated enFlow cartridge increased the temperature to 40 °C at all flow rates.

## Discussion

We compared the warming capacity of two different models of the enFlow intravenous fluid warming cartridge. We tested different flow rates and varying fluid input temperatures using the uncoated and coated fluid warming cartridges. At lower flow rates, both types of enFlow cartridge achieved warming of fluid to the target temperature, regardless of the input temperature. Direct comparisons are difficult due to differences in experimental setups. At higher flow rates, the results showed similar heating performance between the two devices. According to these results, the parylene coating does not appear to negatively impact the heating capacity of the cartridge.

While there were subtle differences in T_out_ between the devices, it is unclear whether these differences are in fact clinically significant. Although 1-2 °C seems trivial at first examination, human temperature is very tightly controlled by the thermoregulatory system. Decreases of only 0.2 °C are associated with homeostatic processes designed to maintain temperature, and a drop in core temperature of 1 °C triggers shivering [8, 30]. While slow infusions of parenteral fluids that are 1 °C below the target temperature may not be sufficient to reduce the core body temperature of the patient, clearly the goal of the health care practitioner is to deliver therapies that will not add further physiologic stress to the body. It would seem logical to select a warmer that delivered fluids at a physiologic temperature. The very low standard deviation in the coated enFlow cartridge suggests a highly consistent temperature output with excellent warming performance.

As seen in Fig. 2, both coated and uncoated cartridges achieved fluid warming at or near the set point of 40 °C at low flow rates. With higher flow rates and lower T_in_, the differences between the set point and T_out_ increased. Resultant temperatures in the uncoated cartridge at 2 ml.min^−1^ fell off by approximately 2 °C, a drop not seen at 10 ml*min^−1^. As we see this decreased temperature only at very slow flow rates, we believe that this decrease in temperature is caused by the cooling of the fluid as it courses from the output of the enFlow device to the temperature sensor. The heated fluids are flowing more slowly through the tube, allowing greater time for heat loss before reaching the sensor [31].

Parylene is a polymer that is applied to electrical circuits and medical devices. It forms a pinhole-free layer at 14 Å. It forms a chemical and moisture barrier at only 10 Å thick, effectively insulating the aluminum heating plates from the infusate [32]. The parylene coating applied to the enFlow cartridge is specified to be between 3 and 6 μm (30,000-60,000 Å). Unlike indirect heating technologies such as microwave, water baths, infrared lighting, and coaxial coils, the enFlow (parylene-coated) cartridge operates based on direct contact of the infusate with the heating unit. The heating unit consists of a corrugated aluminium heating block that is heated internally by a resistive wire. The power to the wire is controlled by a three-step microprocessor algorithm that compares the incoming fluid temperature to the set temperature, triggers adjustable power to the heating block, and measures the outgoing fluid temperature. The power to the heating wire is adjusted to maintain precise control over the temperature. The time from power on to infusate temperature equilibration is less than one second. Published research has confirmed that the parylene coating nearly eliminated the release of aluminium into a variety of infusates, even at operating temperatures and very low infusion rates [26, 33].

Parylene is typically a thermal barrier as well as a chemical and electrical barrier. Parylene has a thermal conductivity rated at 0.082 watts/meters·Kelvin, while water and glass are rated at 1.13 W/m·K and silicone is 163 W/m·K [34]. Despite its apparent thermal insulating capabilities, our results indicate that the thin layer of parylene on the aluminum heating blocks only minimally affected the heating capacity of fluid warmer. The heating capabilities of both the coated and the uncoated fluid warmer were less optimal at high flow rates of 150 and 200 ml/min, although these flow rates are infrequently used in clinical settings. The fluid warmer increased the temperature of the fluid by at least 16.5 degrees using the coldest infusate temperature and at the highest flow rate.

There are several studies evaluating the performance of fluid warmers in the literature [21, 22, 35–37]. The previous studies evaluated the effect of changing one or two variables on the output temperature among different fluid warmers. For example, the studies were descriptive of the effect of changing the flow rate up to 100 ml/min or the initial fluid temperature on one device. Our results at the same flow rates are comparable to these previously published data.

This study has the strength that it evaluated for the first time the impact of a parylene coating on the heating performance of fluid warmers and tested the system in flow rates up to 200 ml/min. Further for the first time, testing was repeated in six enFlow cartridges to confirm the repeatability of the results. Given the current concerns related to aluminum toxicity [27, 38], this study shows that parylene coating does not negatively affect the heating performance of fluid warmers and might be therefore a solution to overcome the concerns about aluminium leaching raised in the literature [24–26] and by the FDA [28].

Notably, we were not able to identify any previous studies of fluid warmer performance at such high flow rates. Infusions at 150 or 200 ml/min are not unusual in the clinical setting and yet the performance of fluid warmers at these infusion rates has not been described. High infusion rates are often used for trauma patients requiring rapid expansion of blood volume due to hemorrhage, for example, or for patients undergoing open abdominal surgery who may lose fluid quickly through evaporation and third-spacing. The coated and uncoated enFlow system was able to warm the infusate towards physiologic temperature even at these high rates.

Our study has some limitations. We only studied the impact of parylene coating on one fluid type, and the efficacy of the device may be different using fluids with higher or lower viscosity such as saline, whole blood, fresh frozen plasma, or infusions high in lipids. Waldmann et al [26] evaluated safety of the parylene coated fluid warmer using a wide variety of fluids but did not specifically address temperature output by fluid type. Patel et al [39] found minimal differences in the ability of warmers to heat crystalloid vs. blood products. Kim et al [40] found significant differences of delivered temperature depending on the length of the tubing and the temperature of the room. We did not vary infusion tubing length in our experiments, nor did we heat different types of fluids. Future studies should evaluate the heating capacity of the coated warming device vs. warmers made by other manufacturers using different types of infusions, varying tubing length, and changes in ambient temperature. Such studies are clinically important but were outside the scope of the current project. We were not able to identify studies to which to compare the higher flow rates tested in our study since other cartridges are not approved for such high rates of flow. Finally, because of differences in setup related to the location of the temperature sensor distal to the warming cartridges, we were not able to directly compare coated vs. uncoated cartridges.

## Conclusion

The parylene coated enFlow cartridges warm the infused fluids at a level and rate that are consistent with those of the uncoated enFlow cartridge. The parylene coating on the aluminum heating blocks of the new enFlow intravenous fluid warmer does not affect its performance compared to its uncoated model. Parylene coating overcomes the recently described aluminum leaching, without reducing the heating capacity of the enFlow system.

## Data Availability

The datasets used and/or analyzed during the current study are available from the corresponding author on reasonable request.
